# Well-Being and the Social Environment of Work: A Systematic Review of Intervention Studies

**DOI:** 10.3390/ijerph14080918

**Published:** 2017-08-16

**Authors:** Kevin Daniels, David Watson, Cigdem Gedikli

**Affiliations:** 1 Employment Systems and Institutions Group, Norwich Business School, University of East Anglia, Norwich NR4 7TJ, UK; David.Watson@uea.ac.uk (D.W.); C.Gedikli@uea.ac.uk (C.G.); 2 What Works for Well-Being Centre, London WC1X 0JL, UK

**Keywords:** employee well-being, job satisfaction, social environments, fairness

## Abstract

There is consistent evidence that a good social environment in the workplace is associated with employee well-being. However, there has been no specific review of interventions to improve well-being through improving social environments at work. We conducted a systematic review of such interventions, and also considered performance as an outcome. We found eight studies of interventions. Six studies were of interventions that were based on introducing shared social activities into workgroups. Six out of the six studies demonstrated improvements in well-being across the sample (five studies), or for an identifiable sub-group (one study). Four out of the five studies demonstrated improvements in social environments, and four out of the five studies demonstrated improvements in indicators of performance. Analysis of implementation factors indicated that the interventions based on shared activities require some external facilitation, favorable worker attitudes prior to the intervention, and several different components. We found two studies that focused on improving fairness perceptions in the workplace. There were no consistent effects of these interventions on well-being or performance. We conclude that there is some evidence that interventions that increase the frequency of shared activities between workers can improve worker well-being and performance. We offer suggestions for improving the evidence base.

## 1. Introduction

There is interest in how work organizations can contribute to improving employee well-being [1,2,3]. Models of employer–employee relations predict that good social environments in the workplace are associated with worker well-being. These models relate to perceived organizational support (POS) [4], organizational climate (OC) [5], social identity (SI) [6], and organizational justice (OJ) [7]. Theoretical perspectives suggest that POS, OC, SI, and OJ may be closely inter-related [4,7,8]. There is evidence that employers can accrue benefits from good social relations between employer and employees [9], and good workplace social environments enable easier implementation of complex organizational change [10,11]. Therefore, it may be the case that interventions to improve POS, OC, SI, and OJ improve employee well-being, and are an important tool for management practice.

However, there is no current synthesis of interventions aimed at improving workplace social environments, representing a significant gap in the literature. Moreover, interventions to improve POS, OC, SI, and OJ may prove complex, and some interventions may produce adverse effects [12]. Therefore, in the present study, we use a systematic review methodology to examine whether interventions that seek to improve various aspects of social environments in organizations, including POS, OC, SI, and OJ, also improve well-being. Because employers may benefit from improved social environments, we also examine effects on performance.

In the present review, we focus on the interventions to improve psychological well-being. Psychological well-being is a core component of well-being [13], and has the following major components [14,15]: (a) subjective assessments of satisfaction, which in the work context can include job satisfaction; (b) hedonic experience, such as a positive affect (e.g., joy, enthusiasm) and the relative absence of a negative affect (e.g., lack of anxiety, feeling calm); and, (c) eudemonic well-being, which includes feelings of autonomy, mastery, personal growth, positive relations with others, purpose in life, and self-acceptance [16]. Our definition of performance is broad, so that we can encompass multiple levels of analysis (e.g., work group or individual), and multiple indicators of performance and performance-relevant outcomes (e.g., productivity, absence, intent to quit).

Before proceeding with our systematic review, we begin by summarizing existing evidence on the four theoretical perspectives that provided the basis for our expectations that interventions to improve workplace social environments will also improve psychological well-being.

### 1.1. Perceived Organizational Support

According to Rhoades and Eisenburger [4], POS refers to workers’ global beliefs that the organization values workers’ contributions, and cares for their well-being. Rhoades and Eisenburger hypothesize that POS relates to well-being and work performance because: POS influences workers’ rewards expectations; POS fulfils workers’ socio-emotional needs related to social identity (see below), and a perception that help is available from the organization if required; and, through social exchange processes, POS encourages worker reciprocity through enhanced performance and more favorable job attitudes.

Three meta-analyses have examined the associations between POS and well-being [4,17], and two papers are based on the same review [18,19]. The meta-analyses found moderate to large meta-correlations between POS and: indicators of well-being (0.28 to 0.61, indicators included affective experience, strain, and job satisfaction); small but statistically reliable to moderate meta-correlations, with indicators of in-role and extra-role performance (0.16 to 0.42); and, small but statistically reliable to moderate meta-correlations with indicators of withdrawal behaviors, including turnover intentions, turnover, and absence (0.11 to 0.49).

### 1.2. Organizational Climate

One meta-analysis [5] and two systematic reviews focused on healthcare contexts [20,21] have examined OC and wellbeing. Gershon et al. [21] defined OC in relation to group and management support. They found only four studies in their review that pertained to well-being (specifically burnout), and these studies were inconclusive. Bronkhorst et al. [20] defined OC as perceptions of the social and interpersonal aspects of work, and included communication, participation, group and leader relations in the global concept of OC. They concluded that OC was related to better mental health, especially for those aspects of OC linked to leader and group relations. Benzer and Horner [5] distinguished between task and relational climates. Task climate refers to goal setting, organizational innovation, and responsiveness. Relational climate refers to work group relations and social recognition and acknowledgement, and therefore relates most closely to social environments in the workplace. Benzer and Horner found that relational climate had statistically reliable and moderate to large meta-correlations with job satisfaction (0.47), perceived stress (−0.25), managers’ ratings of worker performance (0.17), and workers’ ratings of their performance (0.42).

### 1.3. Social Identity

In the workplace, SI refers to the extent to which individuals internalize their membership of a work group or organization into their self-concept [22]. Therefore, SI can reflect a sense of belonging to an organizational community, and embeddedness in the workplace [22]. As a component of SI, organizational identity reflects the extent to which a worker ascribes organizational attributes to their self-concept [6]. High levels of SI may improve well-being through promoting a sense of belonging, providing a sense of purpose, providing a sense of control, providing a sense of self-affirmation, and/or providing a sense that help is available if needed [22].

Two meta-analyses have examined SI at work and well-being [6,22]. Lee et al. [6] found that organizational identification had moderate to strong associations with: job satisfaction (0.45), self-rated in-role performance (0.27), self-rated extra-role performance (0.42), others’ ratings of worker performance (0.19), and others’ ratings of worker extra-role performance (0.29). Steffens et al. [22] found that organizational and work group identification had moderate associations with health (both 0.21), with a stronger association for psychological health (0.23) than physical health (0.16), although the association with physical health is still statistically reliable. Steffens et al. also found that positive indicators of well-being had a stronger association with identity (0.27) than negative indicators such as stress (−0.18).

### 1.4. Organizational Justice

In general terms, OJ refers to the fairness in how workers are treated. It has four major components: distributive justice, which refers to the perceived fairness of outcomes such as pay; procedural justice, which refers to the perceived fairness of how rewards are allocated; interpersonal justice, which refers to the perceptions of politeness and respect in how reward decisions are communicated; and informational justice, which refers to the accuracy and truthfulness of how reward decisions are communicated [7,23]. The four aspects of OJ are strongly inter-related [7,23].

The associations between OJ and well-being have been subject to six meta-analyses [7,23,24,25,26,27], and a systematic review of prospective studies [28]. Across the meta-analyses, there tended to be a small but usually statistically reliable to strong meta-correlations with indicators of well-being (0.10 to 0.49), including indicators of affective experience, job satisfaction, burnout, and health. There were also small, but usually statistically reliable to strong meta-correlations between indicators of job performance (0.10 to 0.69). Ndjaboué et al.’s systematic review [28] of prospective studies found that OJ is consistently related to indicators of mental health and well-being.

### 1.5. Review Questions

Previous reviews have indicated that POS, OC, SI, and OJ are reliably associated with well-being. However, most studies included in the reviews have been cross-sectional and there is little or no evidence from intervention studies in the reviews cited. Therefore, in the present review, we focus on reviewing evidence on interventions that seek to improve the various aspects of social environments in organizations, including POS, OC, SI, and OJ. Our specific questions are:

*(1)*  Do interventions that seek to improve social environments in organizations promote well-being?
*(2)*  Do interventions that seek to improve social environments in organizations improve performance?

In answering the first question, we will also seek to determine whether the interventions work as hypothesized, namely we will examine whether the indicators of social environments in organizations change as a result of the intervention, as well as indicators of well-being. The second question pertains to exploring whether there are gains for employers for implementing interventions to improve the social environments in organizations. Although we did not specifically search for studies with performance as an outcome, where studies reported on performance as well as well-being outcomes, we extracted performance data too. Consistent with recommended practice for systematic reviews of complex well-being interventions, we also examined the interventions for contextual factors that may have affected the implementation of the interventions, or acted to accentuate or attenuate the success of interventions [29].

## 2. Methods

Prior to the review, we developed a review protocol outlining the process for the review. The review team sought input from experienced researchers working in related fields to the review for their advice on relevant search terms to include in the protocol. The protocol was designed according to the best practice PRISMA-P reporting guidelines [30], and registered on PROSPERO, The International Prospective Register for Systematic Reviews.

To determine inclusion/exclusion criteria for studies, we were guided by the PICOS approach (population, intervention, comparators, outcomes, and study design) (see Appendix A) [30,31].

*Population.* We included studies that focused on well-being in the working population in advanced industrial democracies (e.g., EU-15 countries, USA, Australia, Japan). Studies in countries where economic conditions (and therefore work conditions and organizational context) differ markedly from the advanced industrial democracies were excluded. The decision to focus on the advanced industrial democracies was based on institutional factors that may influence well-being in work, including but not limited to: Greater levels of employment protection through legislation; employees’ expectations of their work environment; expectations regarding corporate social responsibility; health and safety legislation; and, widespread and professionalized expertise in occupational health, work psychology, human resource management and other related disciplines in universities and consultancies. Although this bounds the scope of the present review, it does allow for synthesis and practical application of evidence from more homogenous institutional contexts than would be the case if research from other contexts had been included in the review.

*Intervention.* We focused on interventions to change the social environment in work organizations, such as interventions to change POS, OC, SI, or OJ.

*Comparison.* Ideally, we wanted to compare a group or groups who had been subject to a change or intervention in the workplace with a control group who had not. We also included studies where the only comparator was the level of well-being before the intervention.

*Outcomes.* Change in well-being. Studies that investigated performance alongside well-being were also included.

*Study Designs.* Qualitative or quantitative studies that included a longitudinal element were used.

*Other.* Peer reviewed empirical research published in an English/non-English language peer reviewed journal that met the other inclusion criteria as specified above. Articles not containing empirical research were excluded.

### 2.1. Searches

The search terms were developed on the basis of the research questions, consultation with subject matter experts, and the inclusion/exclusion criteria detailed above. The search terms are shown in the Appendix. We did not apply restrictions on date, language, or publication type in the searches, and all citation data were downloaded using reference management software (EndNote X7.4) (Clarivate Analytics, 1500 Spring Garden, Philadelphia, PA, USA). The electronic searches were performed up to 27 September 2016, and targeted the following databases: EconLit, PsycINFO, PubMed Central (PMC), Web of Science, Business Source Complete, and Academic Search Complete.

### 2.2. Study Selection

**Figure 1:**
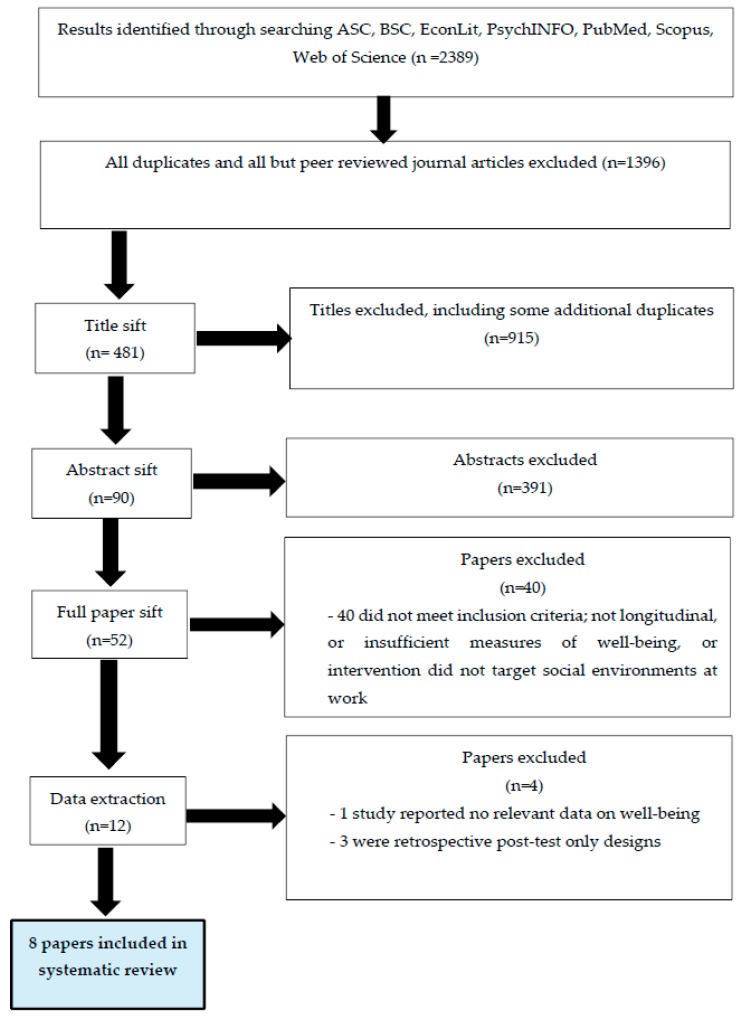
Study selection.

At all stages in the selection of studies, we erred on the side of caution and included studies for the next stage of sifting if there was any doubt whether the study did or did not meet the inclusion criteria. At each stage of sifting, the included papers were sifted independently by two review authors. Any disagreements were recorded, and these were resolved by discussion between the members of the review team. Figure 1 summarizes the process of study selection.

Our first search identified 1396 titles as ‘hits’ to be sifted according to the inclusion criteria. This was performed independently by the three review authors who then met to check reliability. Cohen’s Kappa rating indicated a moderate level of agreement between the reviewers (range 0.59 to 0.65). We then sifted the abstracts according to the inclusion criteria. This was preceded by a pilot sift of 50 abstracts (chosen at random), conducted by all members of the review team to ensure consistency. The abstracts were then sifted independently by the review authors. All disagreements were recorded and these were resolved by discussion. Cohen’s Kappa scores indicated good levels of agreement between the reviewers (range 0.73 to 0.84). All studies that made it through the abstract sift were then assessed as full papers to ascertain whether they did meet the inclusion criteria. Cohen’s Kappa scores indicated moderately good levels of agreement between reviewers at this stage (Kappa was 0.66 in all instances). Out of the original search results, 12 papers made it through to the data extraction phase of the review, which was carried out by all of the review authors. Following the data extraction, four studies were removed from the review because they did not meet inclusion criteria. The decision to exclude these papers was made collectively by the review team following discussion, and after two reviewers had read each discarded paper.

### 2.3. Data Extraction

Data extraction sheets captured detailed information about the nature of the intervention and outcomes. Prior to the data extraction and to ensure consistency, all authors extracted data from one paper as a test case. The papers were then divided between the reviewers for coding. The first author coded all of the papers. To ensure the consistency of coding, all papers were double coded by one of the other authors. Second coders’ comments on papers were incorporated into the first coders’ comments on the data extraction sheets.

Once the data were extracted, the first author synthesized the data extraction sheets into an evidence summary table, which categorized studies into type of intervention, and provided the basis for a narrative summary of evidence around each type of intervention, as well as a summary evidence statement for each type of intervention. Consistent with recommended practice for systematic reviews of complex well-being interventions [29], we then assigned quality gradings for the evidence base underpinning each evidence statement, rather than grading the quality of the individual studies. The final quality grading for evidence was based on recommendations made for reviews of complex interventions targeted at well-being [29]. Snape et al. [29] provide four categories of evidence: “Strong evidence”, in which there is confidence that an intervention has an impact in stated group and context; “Promising evidence”, which suggests an impact may occur but requires further investigation; “Initial evidence”, which requires further investigation and although an effect may occur, there is less confidence than for “promising evidence”; “Evidence not yet strong enough for conclusions”, where there is insufficient evidence to make conclusions. The four categories of evidence are developed from the GRADE approach specified in the Cochrane Centres handbook for quantitative studies [32], and the CERQual approach for qualitative studies [33]. Both approaches have been developed to assess the overall quality of evidence underpinning the evidence findings of the reviews, which is informed by the methodological limitations of individual studies. All three authors met to review the draft evidence summary table and the evidence statements to discuss and reach a consensus on the evidence, how it should be interpreted, and the accuracy of the evidence statements.

Where there was sufficient statistical information presented in papers on a homogenous range of outcomes, we calculated a sample size adjusted meta-analytic Cohen’s *d* to provide a formal statistical test of the effects of interventions. We first calculated Cohen’s *d* for each study, which is the ratio of change in the mean scores from before to after the intervention, divided by the pooled standard deviation of scores from before and after the intervention. To estimate a standard error for meta-analytic Cohen’s *d*, we used the standard error of the raw mean *d* across the five studies.

## 3. Results

Of the eight studies reviewed, six were concerned with the interventions in which people engaged in some sort of shared activity in and around the workplace [34,35,36,37,38,39]. Two were concerned with attempts to improve fairness in the workplace [40,41]. Because the interventions are hypothesized to work through improvements in either social environments in general for the first set of interventions or fairness at work, in the second set of interventions, we also examined whether the interventions improved the presumed mediator of effects on well-being, namely indicators of social environments or fairness. We found sufficient statistical information on a homogenous range of outcomes to calculate a sample size adjusted meta-analytic Cohen’s *d* from only five studies. These were studies of shared activities that measured job satisfaction. In each study, we used the best indicator of overall job satisfaction, which was either an aggregate measure of where information was provided [36,37,38,39], or a single item summative scale [35], where we used an item assessing work enjoyment.

### 3.1. Narrative on Interventions Focused on Shared Activities in and around the Workplace

Because interventions based on shared activities are focused on improving the social environments at work, we also examined whether there was evidence that the social environments in work groups improved subsequent to interventions, as well as well-being and performance. In total, we examined six studies; of which two were non-equivalent control group designs, and four were pre-post-test only with no control group design. Four of the interventions were in healthcare [34,35,38,39], one involved cleaners [34], and one was in the voluntary sector [37]. One intervention was conducted in Sweden [34], one in Denmark [36], one in the UK [38], and three in the US [35,37,39]. Table 1 summarizes the studies.

The interventions included dialogue groups to create shared understandings of how the organization worked [34], team building [35,36], a participative intervention to improve an appraisal system [35], group training accompanied by individual coaching [38], and an intervention focused on mentoring to foster collaboration [39]. The effects of the interventions were assessed in periods ranging from immediately after the intervention, through to 12 months after the intervention. All of the interventions had multiple components and none involved just a short-training course or single shared activity.

Job satisfaction was the only indicator of subjective well-being assessed in five of the studies. In all five studies, job satisfaction was reported to increase as a result of the intervention [35,36,37,38,39]. Only one study reported null effects on well-being [34]. This was the only study to examine other indicators of well-being, which were affective well-being (labelled mental energy by the authors), and work-related exhaustion. In this study, there was a decline in exhaustion for women only, but not for men. There was no change in affective well-being for men or women. Although five out of six studies reported an improvement in job satisfaction, and the other a contingent improvement in another marker of well-being, it should be noted that two studies did not report any formal statistical tests of changes in well-being [35,36].

We were able to calculate a meta-analytic sample size adjusted Cohen’s *d* from the five studies that measured job satisfaction. For the individual studies, the *d* values were 0.53, ([35], with missing data effective n = 118), 0.32 ([36], with missing data effective n = 13), 0.25 ([37], with missing data effective n = 33), 0.89 ([38], with missing data effective n = 19), and 0.17 ([39], with missing data effective n = 58). The sample size adjusted value of the meta-analytic Cohen’s *d* indicated a statistically reliable small to medium effect of the interventions (*d* = 0.42, 95% confidence interval 0.17 to 0.67).

In four out of the five studies, there were improvements in the social environment in the intervention group subsequent to the intervention [35,36,37,39]. These were improvements in group cohesion [33,37], improvements in team working, support and community [36], and improvements in perceived justice and support [37]. Each of the studies that reported improvements in the social environments at work also reported improvements in well-being. The study that found no overall improvements in well-being [34] also found no improvements in the social environment. Therefore, there is evidence that these types of interventions work through the mechanism intended, namely improved social environments at work. One study reported qualitative and quantitative data on the social environment, but did not report formal statistical tests on the quantitative data [36].

In relation to performance, four out of five studies that examined various performance related outcomes reported improvements, which included decreases in markers of intentions to leave the organization [35,39], improved supervisor reports of worker initiative [36], and observer rated performance [38]. One study used interviews with a supervisor to gauge performance increases [36]. Another study found a decrease in intention to leave, but this was not statistically significant [37].

Only one study failed to show any consistent effects [34]. However, the intervention was introduced to an organization that had already been running a similar intervention for several years, and the treatment group included people who had participated in the previous intervention, thus confounding the effect of the intervention.

The positive results of the other studies, which include a statistically reliable aggregated effect across those studies on well-being, may be taken to suggest the evidence is strong. However, the relatively weak designs (no randomized control trials, four out of six studies with no control group) and the absence of formal statistical tests in some studies indicate that the evidence indicates such interventions are “promising” at best. Table 2 provides a summary of the key limitations of each individual study. Moreover, an analysis of the studies which had data on how the interventions were implemented, or where the authors had speculated on reasons for the intervention’s success revealed only one factor that was mentioned in more than one study. This factor was that participants generally had favorable attitudes to the intervention prior to the intervention [35,36,37]. All of the interventions studied used external facilitators or trainers, and took place over several days or months with multiple components. Therefore, our first evidence statement is:

Evidence statement 1: Actions to improve social environments in workplaces through shared activities may improve well-being and a range of performance-relevant outcomes. Such actions require some input external to the workgroups concerned, favorable worker attitudes prior to the intervention, and several different components.

### 3.2. Narrative on Studies Focused on Improving Perceptions of Fairness

For fairness interventions, we examined two randomized control trials. Both had short-term follow-ups (2 weeks or immediately after the intervention). One intervention was a brief intervention, in which advanced notification with justification via email was anticipated to off-set the effects of the introduction of monitoring sales and service workers’ internet activity at work [40]. The other was a more extensive and multicomponent introduction of an appraisal system based on justice theory in the public sector organization [41]. Both studies were conducted in the US. Table 3 summarizes the studies.

In the internet monitoring study [40], advanced notification of internet monitoring had a marginal effect on one dimension of justice perceptions (distributive justice *p* < 0.10). No evidence was provided for changes in job satisfaction, organizational commitment, or intent to leave. However, post-intervention, distributive justice was related to job satisfaction, organizational commitment, and intent to leave. Although this may suggest a mediation effect, any mediation is marginal at best.

In the appraisal study [41], appraises and appraisees reported more favorable attitudes to the appraisal system after changes to the appraisal system had been introduced. Appraisees also reported that they were less likely to leave the organization and had more positive views of their manager. However, there was no change in appraisees’ job satisfaction or motivation to improve performance. Appraisers (managers) also reported more favorable attitudes towards the appraisal system, fewer work problems, and higher job satisfaction.

Another study [37], reviewed as an intervention on shared activities, also reported an attempt to improve the fairness of an appraisal system. This study did also show improvements in job satisfaction and perceptions of justice, although the authors of this study attribute the success of the intervention to the collective approach in addressing a workforce challenge rather than improving a performance appraisal system.

In both fairness studies [40,41], there was some evidence that attempts to improve fairness at work also improved perceptions of fairness. However, in neither study was there consistent nor robust evidence that improved perceptions of fairness realized either improvements in well-being, or improvements in attitudes to work (neither study measured performance directly). Table 4 summarizes the key limitations of both studies. Although both the theory and epidemiological evidence indicate that improved fairness (justice) perceptions also should improve well-being (see Section 1), there is no intervention evidence that demonstrates convincingly how to do so. Therefore, the grading for our evidence statement is rated at ‘not yet strong enough evidence’.

Evidence statement 2: There is not yet strong enough evidence on the effects of interventions that seek to improve well-being through improving the perceptions of fair treatment at work.

## 4. Discussion

The evidence that we have reviewed indicates that activities based on increasing the frequency of shared activities between workers can improve worker well-being and performance via improved social environments at work. The evidence suggests that such activities need to be sustained, have some external facilitation, and have different components for different types of shared activities (for example, externally facilitated training workshops alongside group social activities, internal mentoring programs, and action planning groups around specific organizational issues). It also seems that interventions are more likely to be successful if workers have favorable attitudes towards such interventions. For these types of interventions, there is more extensive and more consistent evidence, albeit with weaker study designs than for fairness interventions. Interventions based on shared activities may be relatively cost-effective when compared to interventions that require changes in organizational processes, such as job redesign or purchase of external services such as employee assistance programs. Although some external facilitation may be required, this might be relatively brief and limited to a few workshops [35,36,38]. Other components of the intervention could include social activities [36,39], internal mentoring programs [39], and communities or practice or action planning groups around specific organizational issues [35,37]. Fairness interventions have strong theoretical and epidemiological support, but there is no strong support from multiple intervention studies.

One surprising finding of the study is that the literature has so few intervention studies related to social environments in the workplace, yet there is a wealth of other research, summarized in several reviews and meta-analyses and well-articulated theories of POS, OC, SI, and OJ. Most of the intervention studies that do exist have been focused primarily on one indicator of well-being, namely job satisfaction. Therefore, there is a need to increase the number of intervention studies, and use a wider range of outcomes, especially given the potential cost effectiveness of interventions based on shared activities. Given the dominance of interventions for healthcare workers in studies of interventions based on shared activities, the absence of studies in manufacturing in this review, and the focus on research in advanced economies, there is a need for studies in a wider range of contexts. However, interventions based on social activities appear to have benefits for well-being across different national contexts, including individualist contexts (UK, US).

As well as the size of the evidence base on interventions, one limitation of the evidence reviewed here is the heterogeneity of the interventions in the different studies. Given both the current size of the evidence base, and the heterogeneity of interventions, we grouped interventions according to high level generic features, rather than specific grouping together of interventions that were very similar. For interventions based on shared social activities, we were able to conclude that such interventions require several components. However, the heterogeneity of the current evidence base means that it is not possible to make any conclusions concerning the best combination of components, or the order in which those components are introduced. One possible exception is that the evidence suggests that workers require favorable attitudes to the intervention prior to the intervention, and therefore actions to gauge the extent of, and/or to inculcate favorable worker attitudes to the shared activities may be required before any other actions.

Similarly, there was a potential heterogeneity for fairness interventions. It is reasonable to propose that fairness around performance appraisal, which has potential implications for wages, may have more salience than fairness around internet monitoring. Even so, there were null results for fairness interventions, which could reflect at least four things. First, the interventions themselves may have been weak influences on justice perceptions. Second, the studies may have had weak statistical power. Third, fairness interventions may have stronger effects if they are targeted at specific and disadvantaged groups (e.g., disabled, ethnic minorities). Fourth, there may have been a contextual effect. The earliest fairness study was published in 1995, and both fairness studies were based in the US. By 1995, most developed economies would have had clear equality and discrimination laws. It may be the case that fairness interventions are best enacted through legislative frameworks that apply across all organizations in a given nation. In this respect, comparative and longitudinal analysis of policy changes in different countries may be informative on the effects of fairness interventions.

Another limitation on the evidence concerns the quality of the studies that comprise the evidence base on shared social activities. Generally, these studies used weak designs, with four out of six studies using a relatively weak pre-post-test only, with no control group designs. Three out of six studies did not report formal statistical tests for some of the effects of the interventions, in spite of using data collected using quantitative ratings scales. Moreover, the studies of shared social activities were not based on explicit theories of well-being, and competing theories of how the interventions may have worked were not examined. That is, no study examined formally whether the effects of the interventions on well-being were mediated by indicators of POS, OC, SI, OJ, or other possible theories. This places a limitation on what can be concluded, in that it is not possible to make explicit the mechanisms that underpin successful interventions. Notwithstanding, it was possible to make some statistical inference across the studies as a whole, with a meta-Cohen’s *d* suggesting a statistically reliable small to medium sized effect for interventions based on shared activities. Consistent results across the studies do indicate that interventions based on shared social activities are promising and require further investigation.

Given the limitations on the size and nature of the evidence base for interventions targeted at improved social relations, it is important for future intervention studies to use stronger designs (e.g., suitably powered randomized controlled designs, non-equivalent control group designs) with long-term follow-ups, strong statistical tests, and a more extensive range of well-being outcomes. Intervention studies could also make explicit use of the realist evaluation approach [42], which focuses researchers on the mechanisms of how interventions work, and whether those mechanisms vary in strength in different contexts, or for different sub-groups exposed to the interventions. Uncovering such mechanisms is theoretically important because intervention studies can provide relatively strong causal inference in ecologically valid settings [43]. Intervention studies require researchers to operationalize specific actions and changes that should alter a theoretical construct, and realist evaluations of interventions can help denote dependencies or redundancies between models [8]. For example, in relation to interventions based on shared activities, it is not clear whether the effects on well-being and better social environments derive from enhanced SI, POS, OC, or some combination thereof.

## 5. Conclusions

There is promising evidence that worker well-being may be improved through a combination of initiatives based on shared social activities. There is insufficient evidence to make any conclusions about the effects on well-being of organizational interventions to improve perceptions of fair treatment at work. Future research on interventions to improve well-being through improving social environments in workplaces requires studies in wider range of contexts, on a more extensive range of well-being outcomes and using stronger study designs.

## Figures and Tables

**Table 1:** Summary of studies on shared social activities.

**Study Reference**	[34]	[35]	[36]
Study design	Pre-post-test only, no control group	Pre-post-test only, no control group	Pre-post-test only, no control group
Nature of data	Quantitative	Quantitative	Mixed methods
Post-intervention follow-up	0 month	12 months	3 weeks
Well-being indicators	Affective well-being (labelled mental energy by authors), work-related exhaustion	Eight facets of job satisfaction	Job satisfaction
Performance relevant indicators	None	Administrative data on staff turnover	Supervisor reports of performance (worker initiative)
Social environment and other relevant indicators	Psychosocial work environment	Group cohesion and group functioning	Psychosocial work environment
Sample size	n = 60 pre-/n = 46 post-intervention	n = 185 pre-/n = 118 post-intervention	n = 14 pre-/n = 13 post-intervention
Study context	Swedish healthcare	US healthcare	Education (cleaning), Denmark
Intervention features	Dialogue groups with 10 doctors and two facilitators, held once a month on 10 occasions over a year. Sessions lasted three hours	Externally facilitated team building. Intervention in each nursing unit consisted of a minimum of 3 one-hour sessions	Eight months long intervention, included Danish lessons for migrants (3 h per week for 6 months), vocational training courses (8 half days), workshops on job satisfaction and teamwork (2 half days), increased frequency of staff meetings, social events. Training delivered by external facilitators
Summary of findings	No change in social climate. No effect on well-being. Exhaustion decreased in women only	Group cohesion increased, staff turnover reduced, seven out of eight facets of job satisfaction increased	Increased social community, support, team working, worker initiative, job satisfaction
**Study Reference**	[37]	[38]	[39]
Study design	Non-equivalent control group	Pre-post-test only, no control group	Non-equivalent control group
Nature of data	Mixed methods	Quantitative	Mixed methods
Post-intervention follow-up	0 months	12 weeks	7 months
Well-being indicators	Job satisfaction	Job satisfaction	Job satisfaction
Performance relevant indicators	Intent to leave profession	Observer rated client support	Intent to stay with the organization
Social environment and other relevant indicators	Psychological climate	None	Group cohesion
Sample size	Intervention group	n = 36 pre-/n = 19 post-intervention	Intervention group
	n = 137 pre-/n = 51 post-intervention		n = 94 pre-/n = 58 post-intervention
	Control group		Control group
	n = 153 single assessment		n = 65 pre-/n = 41 post-intervention
Study context	US voluntary sector	UK healthcare	US healthcare
Intervention features	Participative design team approach to new appraisal system implemented over several months (exact number not provided in the paper), targetted on improving social climate. An external facilitator met with the design team for a minimum of 4 h a month	Client support intervention in three sessions and external facilitation: (i) Class-room training, (ii) interactive training, (iii) 90 min of individual coaching. (i) and (ii) were group based and involved all staff	Mentorship intervention to foster collaboration with other nurses to use evidence based practice. Trainee mentors undertook a two-day workshop. Other components were a lunch workshop, a holiday tea party, interactive lecturers, intranet tutorials
Summary of findings	Improvements in aspects of psychological climate (justice, organizational support) and some facets of job satisfaction (pay, co-workers, benefits). Total job satisfaction improved marginally (*p* < 0.10). Non-significant decrease in intent to leave profession	Improvements in job satisfaction and care given to clients	Improvements in group cohesion, job satisfaction and intent to stay with the organization

**Table 2:** Key limitations of studies on shared social activities.

Study Reference	Key Limitations
[34]	No control group. Intervention confounded with similar previous intervention. Very short follow-up period post-intervention. Single follow-up assessment. Limited generalizability.
[35]	No control group. Limited range of well-being indicators (job satisfaction). No formal statistical tests of impact of intervention on well-being. Single follow-up assessment only. Limited generalizability.
[36]	No control group. Limited range of well-being indicators (job satisfaction). No formal statistical tests of impact of intervention on quantitative indicators. Very short follow-up period post-intervention. Single follow-up assessment. Very small sample size. Limited generalizability.
[37]	Control group non-randomized. Control group limited assessed only once. Limited reporting of qualitative analyses. Limited range of well-being indicators (job satisfaction). Very short follow-up period post-intervention. Single follow-up assessment. Limited generalizability.
[38]	No control group. Limited range of well-being indicators (job satisfaction). Single follow-up assessment only. High attrition rate and very small post-intervention sample size. Limited generalizability.
[39]	Control group not randomized. Limited range of well-being indicators (job satisfaction). Single follow-up assessment. Limited generalizability.

**Table 3:** Summary of studies on fairness perceptions.

Study Reference	[40]	[41]
Study design	Randomized control trial	Randomized control trial
Nature of data	Quantitative	Quantitative
Post-intervention follow-up	2 weeks	0 week
Well-being indicators	Job satisfaction	Job satisfaction
Performance relevant indicators	Organizational commitment, intent to leave	Intent to remain with employer, motivation to improve performance
Social environment and other relevant indicators	Perceptions of distributive, procedural and informational justice	Employee attitudes to appraisal and manager. Manager reports of work problems, satisfaction with appraisal and appraisal distortion
Sample size		Intervention group
	n = 98 responses pre-intervention	n = 51 workers and 51 managers pre-/n = 42 workers and 40 managers post-intervention
	n = 62 responses post-intervention	Control group
	No data provided on numbers in intervention conditions in the paper	n = 41 workers and 41 managers pre-/n = 21 workers and 29 managers post-intervention
Study context	US manufacturing	US public sector
Intervention features	Advanced notice condition received email about internet monitoring prior to introduction (compared notification post-implementation). Justification condition provided a rationale versus no justification. 2*2 design.	Managers and workers trained on new appraisal system, then met three times for employees to clarify performance expectations, give feedback and then conduct formal appraisal. This was a “due process” appraisal based on adequate notice, fair hearing and judgement based on evidence.
Summary of findings	Advanced notification was marginally related to distributive justice (*p* < 0.10). No other effects of the intervention were significant. Distributive justice was signficantly related to job satisfaction, organizational commitment and intent and inversely to turnover post-intervention. Therefore the results suggest a marginal mediated effect of advanced notifcation on job satisfaction, organizational commitment and turnover, but this was not formally tested.	Improvements in workers attitudes towards appraisal, attitudes towards managers and intent to remain with the organization. No changes in job satisfaction or motivation to improve performance. Managers reported fewer appraisal problems, less appraisal distortion, more satisfaction with the appraisal system and more job satisfaction.

**Table 4:** Key limitations of studies on fairness perceptions.

Study Reference	Key Limitations
[40]	Limited range of well-being indicators (job satisfaction). Very short follow-up period post-intervention. Single follow-up assessment. Probably low statistical power due to sample size and complexity of design. Appropriate statistical analysis of well-being outcomes not reported. Limited generalizability.
[41]	Limited range of well-being indicators (job satisfaction). Very short follow-up period post-intervention. Single follow-up assessment. Limited generalizability.

## Appendix A

Search Terms

Population/Sampling keywords: (occupational OR organisational OR organizational OR industrial OR work* OR employ*).

Intervention keywords: (organi*ational_ident* OR *justice OR morale OR organi*ational_climate OR social_climate OR perceived_organi*ational_support OR sense_of_community OR psychological_sense_of_community OR group_engagement* OR social_cohesion OR group_cohesion OR psychological_climate OR organi*ational_community OR communit*_of_practice OR social_ident* OR psychological_contract_breach OR psychological_contract_violation OR team_climate OR social_exchange OR employee_voice OR justice_climate).

Outcome keywords: (stress OR well-being OR wellbeing OR well_being OR mental_health OR mental_ill* OR emotions OR affect* OR mood OR job_satis* OR anxiety OR depress* OR burnout OR engagement OR work_engagement OR employee_engagement OR life_satis* OR job_strain OR psychological_health).

Study design/Methods keywords: (quasi-experiment OR intervention OR field_experiment OR randomised_control_trial OR randomized_control_trial).
